# Hemoglobin albumin lymphocyte and platelet score and all-cause mortality in coronary heart disease: a retrospective cohort study of NHANES database

**DOI:** 10.3389/fcvm.2023.1241217

**Published:** 2023-11-13

**Authors:** Yang Zheng, Yubing Huang, Haitao Li

**Affiliations:** Department of Cardiology, Hainan Province Clinical Medical Center, Hainan General Hospital, Hainan Affiliated Hospital of Hainan Medical University, Haikou, China

**Keywords:** hemoglobin albumin lymphocyte and platelet score, all-cause mortality, coronary heart disease, NHANES, retrospective cohort study

## Abstract

**Aim:**

Anemia, inflammatory status, and malnutrition are all important factors in the prognosis of cardiovascular disease (CVD), and their interactions are also noteworthy. A recent scoring system, the hemoglobin albumin lymphocyte and platelet (HALP) score, combining multi-dimensional metrics, has been used in the prognoses of many diseases except coronary heart disease (CHD). Herein, this study aims to explore the association between HALP score and all-cause mortality in patients with CHD.

**Methods:**

Demographic and clinical data of adult patients with CHD were extracted from the National Health and Nutrition Examination Surveys (NHANES) database from 2003 to 2018 in this retrospective cohort study. Weighted univariate and multivariate COX proportional hazard models were used for covariates screening and exploration of the association between HALP score and all-cause mortality. The evaluation indexes were hazard ratios (ORs) and 95% confidence intervals (CIs). Kaplan-Meier (KM) curve and the receiver operator characteristic (ROC) curve were used to assess the predictive performance of HALP on CHD prognosis. In addition, subgroup analyses of age and congestive heart failure (CHF) were also performed.

**Results:**

Among the eligible patients, 657 died of all-cause mortality. After adjusting for the covariates including age, education level, PIR, marital status, smoking, physical activity, total energy intake, CHF, stroke, hypertension, DM, CKD, cancer or malignancy, monocyte, drug for CVD, treatment for anemia, anticoagulants drug, and adrenal cortical steroids, we found that HALP score was negatively associated with the risk of all-cause mortality [HR = 0.83, 95% CI: (0.74–0.93)]. Compared with patients with high HALP scores, those who had lower HALP scores seemed to have a higher risk of all-cause mortality (all *P *< 0.05). HALP score has a potential predictive value on CHD prognosis with an area under the curve (AUC) of 0.61. Furthermore, in patients aged <65 years, with or without CHF, a lower HALP score was also associated with a higher risk of all-cause mortality (all *P *< 0.05).

**Conclusions:**

HALP score has a potential predictive value on CHD prognosis; however, the causal association between HALP score and mortality in patients with CHD needs further exploration.

## Introduction

Coronary heart disease (CHD) is the most common cardiovascular disease (CVD), accounting for the plurality of worldwide morbidity and mortality (1, 2). CHD causes both healthcare and financial burdens; therefore, finding convenient indicators to predict the risk of mortality in CHD is essential for its prognostic management (3).

Anemia is an important influencing factor in the prognosis of CVD (4–6). A previous study on patients with stable coronary artery disease (CAD) showed that low hemoglobin (HB) was an independent predictor of cardiovascular events and mortality (7). Albumin (ALB), an indicator of the nutritional status of the human body, has been widely used in prognostic studies of CVDs (8). Wada et al. (9) found that pre-procedural serum ALB concentration for percutaneous coronary interventions (PCI) may be a predictor for major adverse cardiac events in patients with CAD. In addition, the inflammatory status of the organism is also an important influencing factor in CVD prognosis. Besides the manifestation of acute inflammation, decreased lymphocytes are strongly associated with CVD prognosis (10), while platelets play an important role in the acute and chronic inflammatory process of CAD (11). Kabat et al. (12) reveal that higher or lower levels of platelets are both linked to an increased risk of mortality from CHD in postmenopausal women.

It is noteworthy that the interaction among anemia, malnutrition, and inflammation may have an impact on disease prognoses (13). Inflammation reduces ALB synthesis, leading to a decrease in serum ALB concentration (14), and high production of cytokines limits iron uptake and inhibits erythrocyte maturation, which can further lead to anemia (15). Also, malnutrition is associated with anemia in patients with CVD (16). Therefore, a scoring system that combines multi-dimensional metrics may be more appropriate for prognostic assessment of patients with CHD. In recent years, a combination of the above indicators with the hemoglobin albumin lymphocyte and platelet score (HALP), has been used in the prognoses of multiple tumors, heart failures (HR), and stroke (17–20). However, no studies have analyzed the association between HALP scores and the risk of all-cause mortality in patients with CHD.

Herein, this study aims to explore the relationship between the HALP score and the risk of all-cause mortality in patients with CHD and assess the predictive performance of the HALP score in order to provide some references for the exploration of a convenient tool to identify high-risk populations with poor CHD prognoses.

## Methods

### Study design and participants

Data on patients with CHD were extracted from the National Health and Nutrition Examination Surveys (NHANES) database from 2003 to 2018 in this retrospective cohort study. NHANES is conducted by the Centers for Disease Control and Prevention (CDC) and the National Center for Health Statistics (NCHS). It uses a complex, multistage stratified probability sample based on selected counties, blocks, households, and persons within households to assess the nutritional and health status of the non-institutionalized population in the United States. Interviews in participants’ homes were conducted by the NCHS-trained professionals, and extensive physical examinations including blood and urine collection were conducted at mobile exam centers (MECs). Details of the study implementation are available online: https://www.cdc.gov/nchs/nhanes/index.htm. In addition, the raw data extracted from the database are shown in the Supplementary Material.

A total of 1,908 adult patients with CHD were initially included. The exclusion criteria were missing information on (1) HALP score, (2) survival, (3) HB, (4) ALB, (5) lymphocytes, and (6) platelets. Finally, 1,633 of them were eligible. The NHANES survey was approved by the institutional review board (IRB) of NCHS. Written informed consent for participation has been obtained before every survey in NHANES. Since the data were deidentified and publicly available, no ethical approval of our IRB was required.

### Diagnosis of coronary heart disease

Diagnosis of CHD was according to the question in the NHANES multiple-choice questionnaire (MCQ160C): “Has a doctor or other health professional ever told you that you had CHD?” The participants who had positive answers were classified as patients with CHD.

### Definition of the hemoglobin albumin lymphocyte and platelet score

In NHANES, a blood sample was obtained through examinations at the MECs, and HALP score-related indexes including serum HB, ALB, lymphocyte, and platelet levels were further analyzed in the laboratory. The HALP score was calculated according to the formula: HB (g/l) × ALB (g/l) × lymphocytes (10^9^/l)/platelets (10^9^/l) due to the fact that the unit of ALB in the database was “g/dl”. We divided the HALP score into four levels according to the quartiles (<37.31, 37.31–51.15, 51.15–69.68, and >69.68) on the basis of previous studies (18, 21).

### Variables collection

We collected potential confounding factors including age, gender, race, education level, marital status, poverty income ratio (PIR), drinking, smoking, physical activity, total energy intake, congestive heart failure (CHF), stroke, hypertension, dyslipidemia, diabetes mellitus (DM), chronic kidney diseases (CKD), liver disease, CVD family history, cancer or malignancy, body mass index (BMI), white blood cell (WBC), monocyte, drug for CVD use, treatment for anemia, anticoagulants drug use, antiplatelet agents use, anticonvulsants use, and adrenal cortical steroids use.

The smoking status was self-reported during the NHANES household interview. Participants who claimed to have smoked fewer than 100 cigarettes in their lives were labeled ‘never smokers’. Smokers were individuals who had smoked more than 100 cigarettes in their lives (22). Drinking status was self-reported based on the question: “Had at least 12 alcohol drinks/1 year?”. Physical activity was defined by the question: “Performing high-intensity or moderate-intensity physical activity” and answered “yes” (23). Hypertension was defined by laboratory inspection, self-reported hypertension, current use of hypotensive drugs, a measured systolic blood pressure (SBP) ≥130 mm Hg or diastolic blood pressure (DBP) ≥90 mm Hg, diagnosed with hypertension, or taking antihypertensive medication. Participants with total cholesterol (TC) ≥200 mg/dl (5.2 mmol/L), triglycerides (TG) ≥150 mg/dl (1.7 mmol/L), low-density lipoprotein cholesterol (LDL-C) ≥130 mg/dl (3.4 mmol/L), HDL-C ≤40 mg/dl (1.0 mmol/L), self-reported hypercholesteremia, or receiving lipid-lowering therapy were identified as dyslipidemia. The diagnosis of DM was fasting blood glucose ≥7.0 mmol/L, glycosylated HB (HbAlc) ≥6.5%, self-reported DM, or receiving hypoglycemic therapy (24). The eGFR was calculated as follows: estimated GFR = 175 × standardized Scr −1.154 × age−0.203 × 1.212 (if Black) × 0.742 (if female), where GFR is expressed as ml/min/1.73 m^2^ of body surface area 41 and Scr is expressed in mg/dl. The total energy intake was calculated according to the first 24-hour dietary recall in the NHANES.

### Outcome and follow-up

The study outcome was all-cause mortality, which was identified using the international classification of diseases, ninth revisions (ICD-9) and ICD-10 codes. In NHANES, the information on death was obtained through the link of the National Death Index (NDI): https://www.cdc.gov/nchs/data-linkage/mortality.htm. The follow-up ended after participants died or up to 31 December 2019.

### Statistical analysis

Measurement data were described using mean ± standard error (mean ± SE) and analysis of variance (ANOVA) for comparison. Enumeration data were expressed as numbers with constituent ratio [*N* (%)] and the chi-square test or Fisher's exact test for the comparison.

We used a set of NHANES special weights: the 2-year cycle of MEC exam weight (wtmec2yr) for statistical analyses because the HALP score was calculated using four laboratory indexes. Weighted univariate COX proportional hazard model analyses were used to screen the covariates. The association between the HALP score and the risk of all-cause mortality was explored using univariate and multivariate COX proportional hazard model analyses. The model was adjusted for age, education level, PIR, marital status, smoking, physical activity, total energy intake, CHF, stroke, hypertension, DM, CKD, cancer or malignancy, monocyte, drug for CVD, treatment for anemia, anticoagulants drug, and adrenal cortical steroids. We also drew a Kaplan–Meier (KM) curve to assess the survival probability of patients at different HALP levels. The receiver operator characteristic (ROC) curves were employed to reflect the predictive performance of HALP on one-year all-cause mortality in patients with CHD and compared with HB, ALB, lymphocyte, and platelet. In addition, subgroup analyses were performed to explore this relationship in patients with different ages and CHF statuses.

The evaluation indexes were hazard ratios (HRs) with 95% confidence intervals (CIs). Two-sided *P *< 0.05 was considered significant. The missing data are shown in Supplementary Table S1, and we employed multiple imputations to handle missing data and minimize bias. Sensitivity analysis of the association between HALP levels and all-cause mortality in CHD patients before and after the imputations of missing data are shown in Supplementary Table S2. Statistical analysis was performed using SAS 9.4 (SAS Institute, Cary, NC, USA) and R version 4.2.2 (Institute for Statistics and Mathematics, Vienna, Austria).

## Results

### Characteristics of the study population

**Figure 1:**
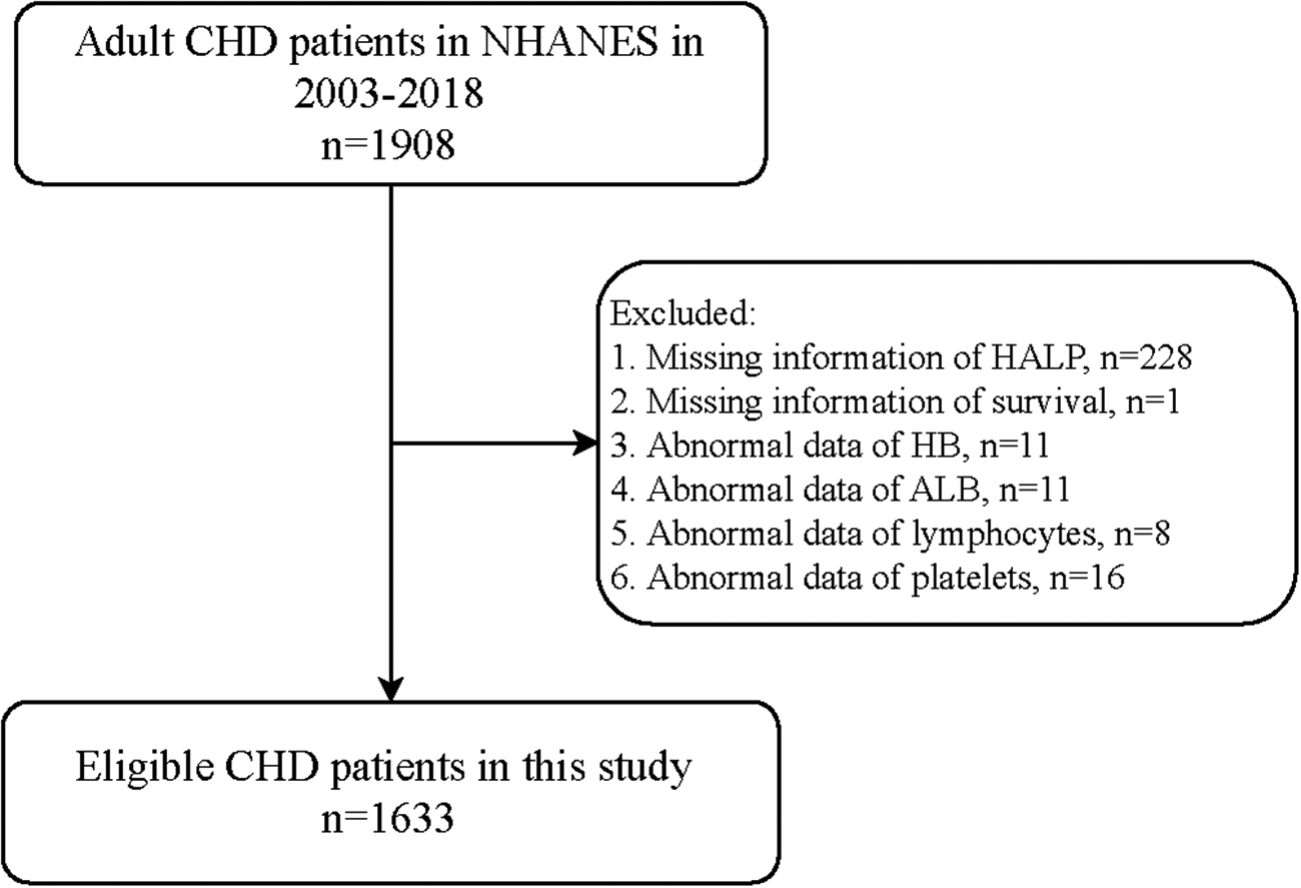
Flowchart of participants screening.

Figure 1 shows the flowchart of the participants screening. We initially included 1,908 adult patients with CHD from the database. Those who had missing information on HALP (*n* = 228), survival (*n* = 1), HB (*n* = 11), ALB (*n* = 11), lymphocyte (*n* = 8), and platelet (*n* = 16) were excluded. Finally, 1,633 of them were eligible.

**Figure 2:**
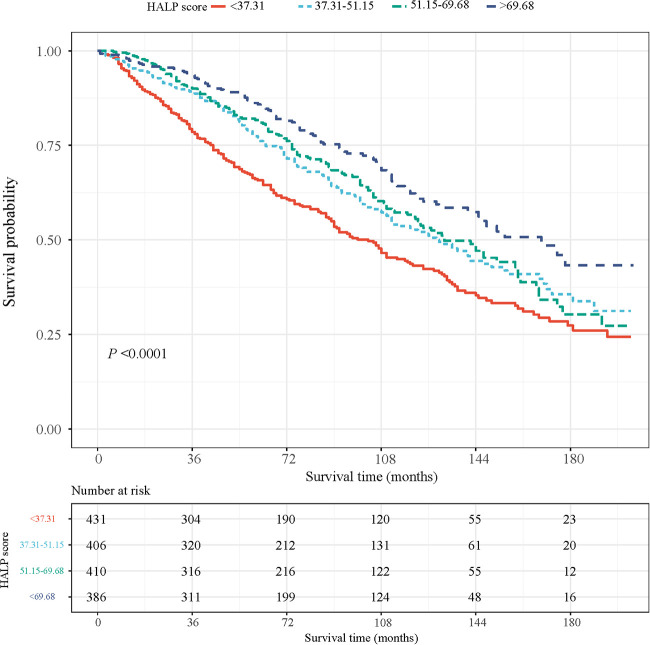
Km curves of the survival probability at different HALP score levels.

After an average of 81 months of follow-up, 657 (33.90%) of the patients died. Their characteristics are shown in Table 1. Most of the patients were ≥65 years old (63.71%) and were men (66.05%). The average HALP score was 56.32 among participants. We compared patients with CHD who had different HALP levels and found that age, gender, smoking, dyslipidemia, CKD, HB, ALB, lymphocyte, platelet, WBC, monocyte, treatment for anemia, anticonvulsants, and adrenal cortical steroids were all significantly different among the four HALP score groups (all *P *< 0.05). Based on these findings, we further plotted the KM curves of the survival probability of patients with CHD at different HALP score levels (Figure 2), which showed that lower HALP scores may be associated with a lower survival probability in patients with CHD.

**Table 1:** Characteristics of CHD patients.

Variable	Total (*n* = 1,633)	HALP scores				Statistics	*P*
		<37.31 (*n* = 431)	37.31–51.15 (*n* = 431)	51.15–69.68 (*n* = 410)	>69.68 (*n* = 386)		
Age, years, *n* (%)						*χ*^2 ^= 10.511	0.015
<65	506 (36.29)	110 (29.40)	115 (33.66)	133 (38.12)	148 (43.98)		
≥65	1,127 (63.71)	321 (70.60)	291 (66.34)	277 (61.88)	238 (56.02)		
Gender, *n* (%)						*χ*^2 ^= 30.189	<0.001
Male	1,116 (66.05)	260 (55.18)	266 (61.15)	287 (69.16)	303 (78.68)		
Female	517 (33.95)	171 (44.82)	140 (38.85)	123 (30.84)	83 (21.32)		
Race, *n* (%)						*χ*^2 ^= 17.611	0.128
Mexican American	166 (3.71)	37 (3.19)	43 (4.24)	41 (4.03)	45 (3.39)		
Other Hispanic	97 (2.39)	21 (1.54)	29 (3.24)	27 (2.35)	20 (2.43)		
Non-Hispanic White	1,051 (81.57)	288 (83.79)	267 (82.26)	259 (79.31)	237 (80.94)		
Non-Hispanic Black	202 (5.77)	61 (6.95)	46 (5.11)	45 (5.16)	50 (5.86)		
Other Race—including multi-racial	117 (6.56)	24 (4.52)	21 (5.16)	38 (9.16)	34 (7.38)		
Education level, *n* (%)						*χ*^2 ^= 2.428	0.876
Less than 11th grade (includes 12th grade with no diploma)	527 (22.92)	130 (22.40)	143 (22.51)	129 (22.90)	125 (23.88)		
High school grade/GED or equivalent	379 (25.70)	107 (24.78)	95 (28.59)	88 (23.28)	89 (26.15)		
Some college or AA degrees and above	727 (51.38)	194 (52.82)	168 (48.90)	193 (53.82)	172 (49.98)		
PIR, mean (S.E)	2.86 (0.06)	2.80 (0.12)	2.80 (0.10)	2.97 (0.12)	2.85 (0.13)	*F *= 0.59	0.626
Marital status, *n* (%)						*χ*^2 ^= 6.521	0.089
Married	956 (62.28)	238 (57.40)	230 (59.73)	244 (63.82)	244 (68.17)		
Other (widowed, divorced, separated, never married, living with a partner)	677 (37.72)	193 (42.60)	176 (40.27)	166 (36.18)	142 (31.83)		
Smoking, *n* (%)						*χ*^2 ^= 15.145	0.019
Never	598 (35.78)	163 (39.75)	165 (41.38)	138 (32.98)	132 (29.03)		
Quitted	765 (46.32)	213 (44.86)	193 (46.74)	191 (45.94)	168 (47.76)		
Smoking	270 (17.89)	55 (15.39)	48 (11.89)	81 (21.08)	86 (23.21)		
Drinking, *n* (%)						*χ*^2 ^= 6.698	0.082
No	445 (24.10)	122 (26.16)	125 (28.22)	105 (22.37)	93 (19.68)		
Yes	1,188 (75.90)	309 (73.84)	281 (71.78)	305 (77.63)	293 (80.32)		
Physical activity, *n* (%)						*χ*^2 ^= 0.376	0.945
No	1,044 (57.34)	276 (58.13)	259 (58.36)	263 (57.26)	246 (55.60)		
Yes	589 (42.66)	155 (41.87)	147 (41.64)	147 (42.74)	140 (44.40)		
Total energy intake, kcal, mean (S.E)	1,924.47 (29.50)	1,868.85 (50.65)	1,876.21 (64.09)	2,010.40 (65.94)	1,942.26 (45.89)	*F* = 1.13	0.341
CHF, *n* (%)						*χ*^2 ^= 1.977	0.577
No	1,127 (73.63)	285 (70.40)	292 (74.87)	283 (75.08)	267 (74.17)		
Yes	506 (26.37)	146 (29.60)	114 (25.13)	127 (24.92)	119 (25.83)		
Stroke, *n* (%)						*χ*^2 ^= 6.247	0.100
No	1,363 (84.95)	351 (84.16)	331 (80.92)	354 (88.42)	327 (86.31)		
Yes	270 (15.05)	80 (15.84)	75 (19.08)	56 (11.58)	59 (13.69)		
Hypertension, *n* (%)						*χ*^2 ^= 4.739	0.192
No	79 (6.49)	20 (6.14)	16 (3.95)	22 (9.15)	21 (6.70)		
Yes	1,554 (93.51)	411 (93.86)	390 (96.05)	388 (90.85)	365 (93.30)		
Dyslipidemia, *n* (%)						*χ*^2 ^= 26.236	<0.001
No	56 (2.55)	28 (6.01)	10 (1.53)	11 (1.48)	7 (1.20)		
Yes	1,577 (97.45)	403 (93.99)	396 (98.47)	399 (98.52)	379 (98.80)		
DM, *n* (%)						*χ*^2 ^= 2.227	0.527
No	956 (60.89)	257 (62.29)	239 (56.92)	243 (63.81)	217 (60.56)		
Yes	677 (39.11)	174 (37.71)	167 (43.08)	167 (36.19)	169 (39.44)		
CKD, *n* (%)						*χ*^2 ^= 18.992	<0.001
No	1,195 (77.79)	281 (70.63)	300 (78.21)	311 (77.24)	303 (85.07)		
Yes	438 (22.21)	150 (29.37)	106 (21.79)	99 (22.76)	83 (14.93)		
Liver disease, *n* (%)						*χ*^2 ^= 2.970	0.396
No	1,531 (93.31)	400 (92.40)	389 (94.58)	386 (94.89)	356 (91.36)		
Yes	102 (6.69)	31 (7.60)	17 (5.42)	24 (5.11)	30 (8.64)		
CVD family history, *n* (%)						*χ*^2 ^= 3.549	0.314
No	1,053 (62.62)	272 (59.31)	261 (65.73)	271 (65.28)	249 (60.16)		
Yes	580 (37.38)	159 (40.69)	145 (34.27)	139 (34.72)	137 (39.84)		
Cancer or malignancy, *n* (%)						*χ*^2 ^= 1.299	0.729
No	1,276 (77.43)	312 (75.64)	322 (76.39)	325 (78.17)	317 (79.50)		
Yes	357 (22.57)	119 (24.36)	84 (23.61)	85 (21.83)	69 (20.50)		
BMI, kg/m^2^, mean (S.E)	30.04 (0.23)	29.70 (0.48)	30.37 (0.38)	29.80 (0.41)	30.29 (0.32)	*F* = 0.69	0.558
HB, g/dl, Mean (S.E)	14.11 (0.06)	13.06 (0.11)	14.07 (0.08)	14.37 (0.08)	14.93 (0.09)	*F *= 54.68	<0.001
ALB, g/l, Mean (S.E)	41.57 (0.10)	40.11 (0.17)	41.24 (0.16)	42.02 (0.18)	42.92 (0.20)	*F* = 52.21	<0.001
Lymphocyte, 1,000 cells/uL, Mean (S.E)	1.93 (0.03)	1.31 (0.03)	1.73 (0.03)	2.07 (0.04)	2.62 (0.06)	*F* = 187.78	<0.001
Platelet, 1,000 cells/uL, Mean (S.E)	216.96 (1.80)	251.56 (3.39)	224.55 (3.52)	209.47 (3.55)	182.33 (3.31)	*F* = 78.04	<0.001
WBC, 1,000 cells/uL, Mean (S.E)	7.42 (0.06)	6.92 (0.15)	7.25 (0.10)	7.41 (0.14)	8.11 (0.12)	*F* = 13.65	<0.001
Monocyte, 1,000 cells/uL, Mean (S.E)	0.62 (0.01)	0.58 (0.01)	0.61 (0.01)	0.63 (0.01)	0.67 (0.01)	*F* = 7.87	<0.001
HALP, Mean (S.E)	56.32 (0.97)	27.51 (0.39)	44.39 (0.26)	59.28 (0.34)	94.01 (1.95)	*F* = 1,749.34	<0.001
Drug for CVD, *n* (%)						*χ*^2 ^= 2.176	0.537
No	186 (13.49)	38 (11.22)	40 (12.27)	50 (15.16)	58 (15.30)		
Yes	1,447 (86.51)	393 (88.78)	366 (87.73)	360 (84.84)	328 (84.70)		
Treatment for anemia, *n* (%)						*χ*^2 ^= 36.371	<0.001
No	1,505 (93.05)	366 (85.50)	381 (93.65)	394 (96.33)	364 (96.69)		
Yes	128 (6.95)	65 (14.50)	25 (6.35)	16 (3.67)	22 (3.31)		
Anticoagulants drug, *n* (%)						*χ*^2 ^= 5.268	0.153
No	1,422 (88.32)	358 (84.73)	354 (89.17)	373 (91.37)	337 (88.00)		
Yes	211 (11.68)	73 (15.27)	52 (10.83)	37 (8.63)	49 (12.00)		
Antiplatelet agents, *n* (%)						*χ*^2 ^= 3.550	0.314
No	1,182 (71.17)	307 (67.32)	288 (69.97)	300 (72.52)	287 (74.85)		
Yes	451 (28.83)	124 (32.68)	118 (30.03)	110 (27.48)	99 (25.15)		
Anticonvulsants, *n* (%)						*χ*^2 ^= 11.426	0.010
No	1,424 (87.86)	359 (84.97)	380 (93.73)	354 (87.49)	331 (85.26)		
Yes	209 (12.14)	72 (15.03)	26 (6.27)	56 (12.51)	55 (14.74)		
Adrenal cortical steroids, *n* (%)						*χ*^2 ^= 12.983	0.005
No	1,554 (94.72)	397 (91.02)	385 (94.48)	394 (95.06)	378 (98.32)		
Yes	79 (5.28)	34 (8.98)	21 (5.52)	16 (4.94)	8 (1.68)		
All-cause mortality, *n* (%)						*χ*^2 ^= 27.075	<0.001
Survival	976 (66.10)	206 (56.99)	236 (63.06)	257 (67.03)	277 (77.29)		
Mortality	657 (33.90)	225 (43.01)	170 (36.94)	153 (32.97)	109 (22.71)		
Follow-up time, months, Mean (S.E)	81.59 (2.37)	79.48 (4.27)	83.11 (4.16)	82.17 (3.42)	81.59 (3.95)	*F* = 0.14	0.938

*F*, analysis of variance (ANOVA); *χ*^2^, chi-square test; CHD, coronary heart disease; HALP, hemoglobin albumin lymphocyte and platelet; PIR, poverty income ratio; S.E, standard error; CHF, congestive heart failure; DM, diabetes mellitus; CKD, chronic kidney disease; CVD, cardiovascular disease; BMI, body mass index; HB, hemoglobin; ALB, albumin; WBC, white blood cell.

### Association between HALP scores and all-cause mortality

Supplementary Table S3 shows the covariates related to all-cause mortality, including age, education level, PIR, marital status, smoking, physical activity, total energy intake, CHF, stroke, hypertension, DM, CKD, cancer or malignancy, monocyte, drug for CVD, treatment for anemia, anticoagulants drug, and adrenal cortical steroids (all *P *< 0.05).

Then, we explored the relationship between HALP score and all-cause mortality in patients with CHD (Table 2). After adjusting for the covariates, we found that the HALP score was negatively associated with the risk of all-cause mortality [HR = 0.83, 95% CI: (0.74–0.93)]. Compared with patients whose HALP scores were >69.68, those who had HALP scores ≤69.68 seemed to have a high risk of all-cause mortality [HALP score of 51.15–69.68: HR = 1.36, 95% CI: (1.01–1.84); HALP score of 37.31–51.15: HR = 1.41, 95% CI: (1.07–1.85); and HALP score <37.31: HR = 1.64, 95% CI: (1.24–2.17)].

**Table 2:** Association between HALP scores and all-cause mortality.

Variables	Crude model		Adjusted model^a^	
	HR (95% CI)	*P*	HR (95% CI)	*P*
HALP	0.78 (0.69–0.90)	<0.001	0.83 (0.74–0.93)	0.001
HALP Levels				
<37.31	1.94 (1.42–2.65)	<0.001	1.64 (1.24–2.17)	<0.001
37.31–51.15	1.59 (1.21–2.09)	0.001	1.41 (1.07–1.85)	0.014
51.15–69.68	1.45 (1.07–1.96)	0.017	1.36 (1.01–1.84)	0.049
>69.68	Ref		Ref	

HALP, hemoglobin albumin lymphocyte and platelet; HR, hazard ratio; CI, confidence interval; Ref, reference.

^a^ Adjusted for age, education level, PIR, marital status, smoking, physical activity, total energy intake, CHF, stroke, hypertension, DM, CKD, cancer or malignancy, monocyte, drug for CVD, treatment for anemia, anticoagulants drug, and adrenal cortical steroids.

**Figure 3:**
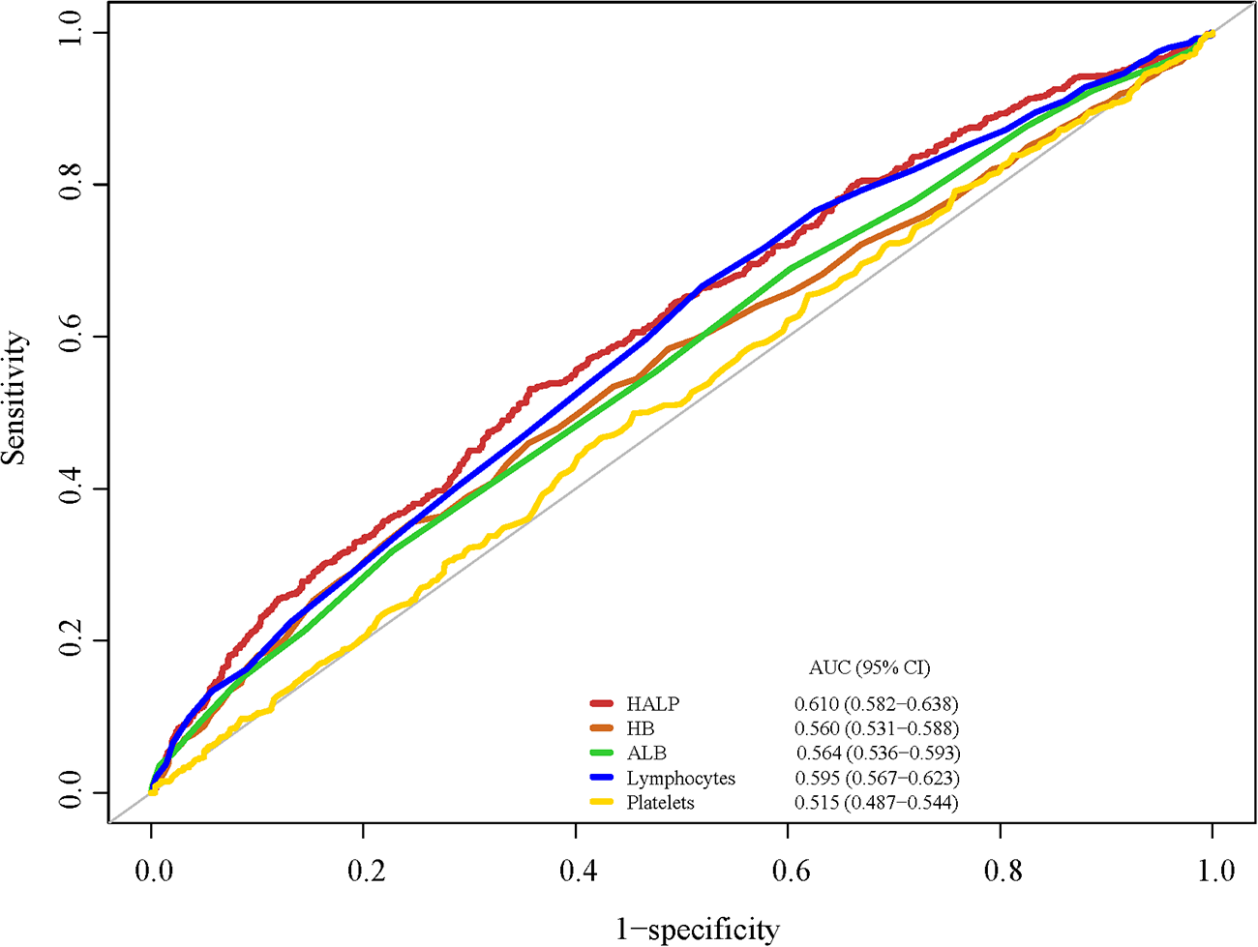
The predictive performance of HALP, HB, ALB, lymphocyte, and platelet on one-year all-cause mortality.

Since the HALP score was calculated by HB, ALB, lymphocyte, and platelet, we compared the predictive performance of HALP and the above four serum indexes on all-cause mortality (Figure 3). The ROCs showed that the HALP score has an AUC of 0.610 and that of HB, ALB, lymphocyte, and platelet were 0.560, 0.564, 0.595, and 0.515, respectively. The predictive values of these indexes are shown in Table 3. HALP score had a sensitivity of 0.510 and specificity of 0.654 on CHD prognosis, indicating a potential predictive value.

**Table 3:** Predictive values of HALP scores on all-cause mortality.

Variables	AUC (95% CI)	Accuracy (95% CI)	Sensitivity (95% CI)	Specificity (95% CI)	PPV (95% CI)	NPV (95% CI)
HALP	0.610 (0.582–0.638	0.596 (0.572–0.620)	0.510 (0.472–0.548)	0.654 (0.624–0.684)	0.498 (0.460–0.536)	0.665 (0.635–0.694)
HB	0.560 (0.531–0.588)	0.594 (0.570–0.618)	0.356 (0.320–0.393)	0.754 (0.727–0.781)	0.494 (0.449–0.539)	0.635 (0.607–0.663)
ALB	0.564 (0.536–0.593)	0.590 (0.565–0.614)	0.318 (0.282–0.354)	0.773 (0.746–0.799)	0.485 (0.438–0.532)	0.627 (0.600–0.655)
Lymphocytes	0.595 (0.567–0.623)	0.555 (0.531–0.580)	0.667 (0.631–0.703)	0.481 (0.449–0.512)	0.463 (0.432–0.495)	0.682 (0.647–0.716)
Platelets	0.515 (0.487–0.544)	0.495 (0.470–0.519)	0.489 (0.450–0.527)	0.499 (0.468–0.530)	0.396 (0.363–0.430)	0.592 (0.558–0.625)

HALP, hemoglobin albumin lymphocyte and platelet; AUC, area under the curve; CI, confidence interval; PPV, positive predictive value; NPV, negative predictive value; HB, hemoglobin; ALB, albumin.

### Relationship between HALP score and all-cause mortality in age and CHF subgroups

We further explored this association in age and CHF subgroups (Table 4). In patients who are <65 years old, we have not found an association between the HALP score and the risk of all-cause mortality (all *P *> 0.05). Nevertheless, those who were ≥65 years old with a HALP score of <37.31 may have an increased risk of all-cause mortality [HR = 1.86, 95% CI: (1.38–2.51)]. Furthermore, patients with [HR = 2.00, 95% CI: (1.29–3.10)] or without [HR = 1.48, 95% CI: (1.01–2.17)] CHF having HALP scores less than 37.31 both had an increased risk of all-cause mortality.

**Table 4:** Association between HALP scores and all-cause mortality in age and CHF subgroups.

Variables	HR (95% CI)	*P*
Age <65 (*n* = 506)		
HALP	0.96 (0.76–1.22)	0.747
HALP Levels		
<37.31	0.82 (0.38–1.77)	0.616
37.31–51.15	1.84 (0.96–3.50)	0.065
51.15–69.68	1.80 (0.98–3.28)	0.057
>69.68	Ref	
Age ≥65 (*n* = 1,127)		
HALP	0.78 (0.69–0.88)	<0.001
HALP Levels		
<37.31	1.86 (1.38–2.51)	<0.001
37.31–51.15	1.37 (1.02–1.86)	0.039
51.15–69.68	1.24 (0.91–1.67)	0.167
>69.68	Ref	
CHF (*n* = 506)		
HALP	0.79 (0.68–0.91)	0.002
HALP Levels		
<37.31	2.00 (1.29–3.10)	0.002
37.31–51.15	1.51 (0.97–2.34)	0.067
51.15–69.68	1.41 (0.93–2.15)	0.105
>69.68	Ref	
Non-CHF (*n* = 1,127)		
HALP	0.87 (0.74–1.01)	0.072
HALP Levels		
<37.31	1.48 (1.01–2.17)	0.042
37.31–51.15	1.30 (0.93–1.84)	0.125
51.15–69.68	1.31 (0.89–1.92)	0.176
>69.68	Ref	

HALP, hemoglobin albumin lymphocyte and platelet; CHF, congestive heart failure; HR, hazard ratio; CI, confidence interval. Age subgroup adjusted for education level, PIR, marital status, smoking, physical activity, total energy intake, CHF, stroke, hypertension, DM, CKD, cancer or malignancy, monocyte, drug for CVD, treatment for anemia, anticoagulants drug, and adrenal cortical steroids; CHF subgroup adjusted for age, education level, PIR, marital status, smoking, physical activity, total energy intake, stroke, hypertension, DM, CKD, cancer or malignancy, monocyte, drug for CVD, treatment for anemia, anticoagulants drug, and adrenal cortical steroids.

## Discussion

In this study, we explored the relationship between HALP scores and all-cause mortality in patients with CHD. The results showed that HALP scores were negatively associated with all-cause mortality. Patients with CHD who had lower HALP scores seemed to have a higher risk of all-cause mortality. We also assessed the performance of the HALP score on CHD prognosis, which indicated that the HALP score had a potential predictive value. In addition, the association between a HALP score of <37.31 and a high risk of all-cause mortality was also found in patients who were ≥65 years old or with/without CHF.

HALP score has been recently defined as a combination score system that can predict the patient's prognosis much better than a single index. HALP score assesses both the immune system and the nutritional status of the patients and has been applied in the prediction of various diseases’ prognoses such as cancers (17, 19, 20, 25). However, studies on the predictive value of HALP score in CHD prognosis have been scarce. Tian et al. (18) suggested that an increased HALP score was related to a decreased risk of mortality within 90 days and 1 year in consecutive acute ischemic stroke (AIS) patients. Li et al. (26) found that the HALP score may be a potential protective marker in acute and subacute cerebral venous sinus thrombosis (CVST) patients, and the HALP model had a potentially prognostic value. In the current study, we similarly found that HALP score was negatively associated with all-cause mortality in patients with CHD, and there may be a potential value of HALP score on CHD prognosis. CHD has been characterized as a chronic immunoinflammatory disease fueled by lipids and potentially modifiable risk factors such as systemic inflammation, DM, high-density lipoprotein (HDL), plasma triglycerides (TG), remnant lipoproteins (RLP), and vascular endothelial dysfunction (ED) (2). A retrospective study on plasma proteins and CVD mortality in patients with chronic CHD showed that myocardial strain–dysfunction–hypertrophy–fibrosis, myocyte death and apoptosis, kidney injury, hemodynamic stress, renin–angiotensin system (RAS) activation, oxidative stress and inflammation, and angiogenesis and vascular cell proliferation (HGF) are important mechanisms associated with CVD mortality (27). Additionally, nutrition also plays a primary role in cardiovascular function. In patients with ischemic coronary disease and heart failure, high or low BMI were both associated with cardiac metabolism and function (28). Based on previous evidence, we indicated that the HALP score can comprehensively assess inflammation levels, and the nutritional status of the organism may be a convenient and detailed predictor for CHD prognosis. However, the current study could not find any large predictive advantage of the HALP score over the single indicators, and future prospective cohort studies are needed to confirm its potential predictive value.

According to the formula for HALP score calculation, the serum HB, ALB, and lymphocyte concentration increased along with the HALP score rising, while that of platelet decreased. We found a potential value of HALP score on CHD prognosis with a little bit higher AUC than the other four single indexes. The low HB often coexisted with inflammation. In a large stable CAD population, low HB was an independent predictor of mortality, cardiovascular events, and major bleeds (7). Inflammation can trigger the process of thrombosis and platelets participate in the adhesion, release reaction, and aggregation of it (29). Lymphocytes play an important role in the elimination and repair of inflammation, and lymphocyte counts have been reported to be an independent significant risk factor to have predictive value for CHD and 1-year major adverse cardiac events (30). ALB has a protective effect due to its antagonism of oxidation, thrombosis, and leukocyte adhesion (31, 32). Low serum ALB was also independently related to CHD (33). HALP score is a cost-effective, simple parameter that can easily assess the inflammation–nutritional status. The finding of the HALP score's predictive value was significant because the instant inflammation–nutritional status assessment may help clinicians assess prognosis and formulate appropriate treatment plans in patients with CHD.

We also explored the association between HALP score and all-cause mortality in patients with different ages and CHF statuses. In NHANES, most of the CHD patients were ≥65 years old (63.71%), and among them, those who had lower HALP scores seemed to have higher hazards of all-cause mortality. Aging is associated with CHD, and the potential mechanisms might be metabolism disorders and DNA methylation (34). Si et al. (35) identified that DNA methylation age was associated with the incidence of CHD. Gutierrez-Mariscal et al. (36) suggested that patients with CHD who were ≥60 years old were less metabolic flexible and showed the same risk of suffering from carotid atherosclerosis as those who with metabolic disease. Moreover, we also found that patients with or without CHF had HALP scores that were both associated with all-cause mortality. Kocaoglu S et al. (37) compared the efficiency of the HALP score and the modified HALP score in predicting mortality in patients with acute heart failure (AHF) but found that the classical HALP score was not adequate for predicting early and late prognosis of AHF patients. The most common comorbidities of AHF were arterial hypertension, dyslipidemia, and CAD (38). However, the mechanisms of the relationship between HALP scores and all-cause mortality in CHD patients of different ages and CHF statuses have not been clear and need further exploration. The results of the current study suggested that clinicians should focus on the HALP scores of CHD patients who are ≥65 years old and with/without CHF for further adjustment of their treatment strategies and enhance real-time testing to reduce the risk of mortality.

This was a retrospective cohort study that may provide some references for the exploration of causal associations between HALP scores and CHD prognosis. Our study extracted data from the NHANES database with a well-represented population selected through a multistage complex sampling. In addition, the four indicators that make up the HALP score are all easy to measure and have good clinical application value. However, there are some limitations in the current study. Because of the retrospective characteristic nature of this study, the inherent selection bias of this design type cannot be avoided. There was a limited number of study sample size; however, sufficient follow-up time has ensured the number of outcome cases. Further validation and refinement are needed to overcome these limitations and enhance the practicality and reliability of the HALP score in CHD prognosis.

## Conclusion

This study provided some references for the potential value of the HALP score for CHD prognosis but further exploration of the practicality and reliability of the HALP score is still needed.

## Data Availability

Publicly available datasets were analyzed in this study. This data can be found here: NHANES database, https://wwwn.cdc.gov/nchs/nhanes/.
