# Supplementary material for: Hemoglobin albumin lymphocyte and platelet score and all-cause mortality in coronary heart disease: a retrospective cohort study of NHANES database
Source: Front Cardiovasc Med. 2023 Nov 13;10:1241217. doi: 10.3389/fcvm.2023.1241217 (PMC10679332; doi:10.3389/fcvm.2023.1241217)
Supplement: Supplementary file 1 [file Table1.docx]

**Table S1. Missing data and their proportions**

| Variables | N (%) |
| --- | --- |
| PIR | 125 (7.65) |
| Total energy intake | 89 (5.45) |
| BMI | 43 (2.63) |
| Education level | 2 (0.12) |
| Smoking | 1 (0.06) |
| Drinking | 105 (6.43) |
| CVD family history | 61 (3.74) |
| Treatment for anemia | 3 (0.18) |

PIR: poverty income ratio, BMI: body mass index, CVD: cardiovascular disease.

**Table S2.** **Association between HALP levels and all-cause mortality in CHD patients before and after imputation of missing data**

| Variables | Before imputation | | After imputation | |
| --- | --- | --- | --- | --- |
|  | HR (95% CI) | *P* | HR (95% CI) | *P* |
| HALP | 0.83 (0.73-0.94) | 0.003 | 0.83 (0.74-0.93) | 0.001 |
| HALP Levels |  |  |  |  |
| <37.31 | 1.65 (1.20-2.26) | 0.002 | 1.64 (1.24-2.17) | <0.001 |
| 37.31-51.15 | 1.35 (0.98-1.84) | 0.063 | 1.41 (1.07-1.85) | 0.014 |
| 51.15-69.68 | 1.37 (0.98-1.90) | 0.065 | 1.36 (1.01-1.84) | 0.049 |
| >69.68 | Ref |  | Ref |  |

HALP: hemoglobin albumin lymphocyte and platelet, CHD: coronary heart disease, HR: hazard ratio, CI: confidence interval, Ref: reference

**Table S3. Potential confounding factors of all-cause mortality**

| Variables | All-cause mortality | |
| --- | --- | --- |
|  | HR (95% CI) | *P* |
| Age |  |  |
| <65 | Ref |  |
| ≥65 | 3.36 (2.58-4.38) | <0.001 |
| Gender |  |  |
| Male | Ref |  |
| Female | 1.12 (0.90-1.39) | 0.308 |
| Race |  |  |
| Mexican American | Ref |  |
| Other Hispanic | 0.79 (0.41-1.50) | 0.462 |
| Non-Hispanic White | 1.37 (0.95-1.96) | 0.087 |
| Non-Hispanic Black | 0.94 (0.62-1.43) | 0.781 |
| Other Race - including multi-racial | 1.34 (0.74-2.43) | 0.338 |
| Education level |  |  |
| Less than 11^th^ grade (includes 12^th^ grade with no diploma) | Ref |  |
| High school grade / GED or equivalent | 0.84 (0.66-1.08) | 0.182 |
| Some college or AA degree and above | 0.67 (0.53-0.85) | 0.001 |
| PIR | 0.80 (0.76-0.85) | <0.001 |
| Marital status |  |  |
| Married | Ref |  |
| Other (widowed, divorced, separated, never married, living with partner) | 1.74 (1.42-2.14) | <0.001 |
| Smoking |  |  |
| Never | Ref |  |
| Quitted | 1.23 (1.01-1.50) | 0.046 |
| Smoking | 0.93 (0.66-1.30) | 0.651 |
| Drinking |  |  |
| No | Ref |  |
| Yes | 0.81 (0.65-1.03) | 0.083 |
| Physical activity |  |  |
| No | Ref |  |
| Yes | 0.45 (0.35-0.56) | <0.001 |
| Total energy intake | 0.99 (0.99-0.99) | 0.006 |
| CHF |  |  |
| Yes | Ref |  |
| No | 0.48 (0.39-0.58) | <0.001 |
| Stroke |  |  |
| Yes | Ref |  |
| No | 0.48 (0.38-0.61) | <0.001 |
| Hypertension |  |  |
| No | Ref |  |
| Yes | 2.57 (1.38-4.81) | 0.003 |
| Dyslipidemia |  |  |
| No | Ref |  |
| Yes | 0.80 (0.52-1.24) | 0.318 |
| DM |  |  |
| No | Ref |  |
| Yes | 1.63 (1.38-1.92) | <0.001 |
| CKD |  |  |
| No | Ref |  |
| Yes | 2.73 (2.29-3.24) | <0.001 |
| Liver disease |  |  |
| Yes | Ref |  |
| No | 0.84 (0.56-1.26) | 0.385 |
| CVD family history |  |  |
| No | Ref |  |
| Yes | 0.96 (0.79-1.16) | 0.646 |
| Cancer or malignancy |  |  |
| Yes | Ref |  |
| No | 0.65 (0.52-0.82) | <0.001 |
| BMI | 0.98 (0.97-0.99) | 0.130 |
| WBC | 1.06 (1.01-1.11) | 0.054 |
| Monocyte | 3.51 (2.28-5.42) | <0.001 |
| Drug for CVD |  |  |
| No | Ref |  |
| Yes | 2.67 (1.83-3.89) | <0.001 |
| Treatment for anemia |  |  |
| No | Ref |  |
| Yes | 1.75 (1.16-2.65) | 0.008 |
| Anticoagulants drug |  |  |
| No | Ref |  |
| Yes | 2.13 (1.68-2.70) | <0.001 |
| Antiplatelet agents |  |  |
| No | Ref |  |
| Yes | 0.88 (0.68-1.14) | 0.339 |
| Anticonvulsants |  |  |
| No | Ref |  |
| Yes | 1.28 (0.96-1.71) | 0.088 |
| Adrenal cortical steroids |  |  |
| No | Ref |  |
| Yes | 2.82 (1.80-4.40) | <0.001 |

HR: hazard ratio, CI: confidence interval, Ref: reference, CHF: congestive heart failure, DM: diabetes mellitus, CKD: chronic kidney disease, CVD: cardiovascular disease, BMI: body mass index, WBC: white blood cell.
